# National Variations in the Work-Up, Investigation, and Surgical Management of Ductal Carcinoma In Situ of the Breast across Canadian Surgeons

**DOI:** 10.3390/curroncol28020130

**Published:** 2021-03-29

**Authors:** Ryerson Seguin, Lashan Peiris

**Affiliations:** Division of General Surgery, University of Alberta, Edmonton, AB T6G 2R3, Canada

**Keywords:** DCIS, variation, guideline, SLNB

## Abstract

Variation in the management of Ductal Carcinoma In Situ (DCIS) of the breast occur at both national and international levels. The aim of this study is to determine the degree of, and reasons behind, this variation in the workup and treatment of DCIS among Canadian surgeons. We developed a 35-question survey involving the pre-, peri, and post-operative management of DCIS using SurveyMonkey^®^. The survey was sent out via email and responses were analyzed using SurveyMonkey^®^ and Microsoft Excel. 51/119 (43%) of the Canadian General Surgeons contacted participated in this study. Some variation was observed in the utilization of pre-operative imaging with 29/48 (60%) surgeons routinely using ultrasound. Perceived contraindications to breast conserving therapy also varied with multicentricity (54%) and the presence of diffuse microcalcifications (13%). Nearly all respondent’s (98%) patients had access to immediate breast reconstruction following a mastectomy but 14/48 (29%) of respondents’ patients were required to travel a mean distance of 300 km to undergo the procedure. Substantial variation was also seen during follow-up with half (52%) of surgeons following up patients for >1 month in their surgical clinic. There is considerable variation in the management of DCIS among Canadian Surgeons. The present study indicates the need for pan-Canadian, evidence-based guidelines to ensure a standardized management strategy for patients with DCIS.

## 1. Introduction

Advances in breast screening techniques have led to a notable increase in the discovery of precancerous lesions of the breast [1,2]. This is especially evident for Canadians as breast cancer is projected to be the second most commonly diagnosed cancer [3]. In particular, mammographic advances have meant that Ductal Carcinoma In Situ (DCIS) represents 20–25% of newly diagnosed breast cancers [4]. The majority of DCIS (90%) is detected by the presence of microcalcifications on mammography requiring image-guided biopsy for formal pathological diagnosis. In a minority of cases (10% of the time), a palpable mass and/or nipple discharge representing DCIS can be felt/seen during physical exam. In these rare cases, there is often no evidence of microcalcifications on mammography [5].

While DCIS is non-invasive, studies have shown that 14–53% of patients with this disease can progress to an invasive form of breast cancer [6].

Regrettably, there are no current methods to assess if or when the disease will progress. This lack of certainty regarding the outcome of DCIS, as well as an increasing incidence (due to screening advances), has led to a rise in surgical treatment of this disease—to maximize the perceived risk-reduction of either an in-situ or, more importantly, an invasive recurrence [1,7]. This practice continues despite ongoing prospective trials investigating whether some of these lesions can be managed with surveillance-alone [8]. These trials have led to studies investigating variation in the current management of DCIS [7].

As first-line treatment, the National Comprehensive Cancer Network (NCCN) guidelines recommend performing a lumpectomy with whole breast radiation therapy in order to achieve disease-free margins (NCCN Category 1). Additional options for treatment include a total mastectomy—for large volume microcalcifications in relation to total breast volume if breast conserving therapy is contraindicated (e.g., prior radiation therapy) or if disease free margins are not possible with a lumpectomy (NCCN Category 2A) or, finally, for patient preference. In the era of Oncoplastic Breast Surgery (OPBS) and Partial Breast Irradiation (PBI), some of these old dogmas are being challenged. For those patients who are categorized as low risk, a lumpectomy without radiation therapy—(NCCN Category 2B) remains a viable option.

Of those patients treated with Breast Conserving Therapy (BCT), there is a <10% risk of local recurrence, with up to 50% of those recurrences representing an invasive form of the disease [2]. With regards to margins, it is uniformly accepted that negative margins decrease the likelihood of local recurrence of the disease. However, recent consensus guidelines have established a minimum margin width for DCIS [9]. Most guidelines base their recommendations on a meta-analysis by Houssami et al., who determined that a minimum margin of 2 mm after BCT for DCIS provides optimal local control, without compromising cosmesis or patient safety due to repeat revisional surgeries. Extending the width of a negative margin to achieve greater than 2 mm had no significant impact regarding local recurrence rates and is therefore not recommended [9].

The aim of this study is to observe variations in practice amongst Canadian general surgeons in the management of DCIS. Adherence to national and international guidelines will be assessed by generating a snapshot of current surgical practice within Canada. These variations in the workup, management, and follow-up may help guide the proposal for a pan-Canadian, evidence-based guideline regarding the work-up and management of DCIS and thus improve the outcome of patients diagnosed with this disease.

## 2. Methods

With Research Ethics Board approval (HREBA.CC-20-0155), we created a questionnaire-based survey using an online survey tool (SurveyMonkey^®^, Ottawa, ON, Canada) and distributed it via email to 119 general surgeons with a known sub-specialty interest in breast surgical oncology across Canada. Questions were designed after a literature review of current practice and the survey was made available for a period of 6 weeks from April 2020 to May 2020. Participants were identified/contacted from every province but subsequently selected based on a demonstrated interest in breast surgery. Identification of surgeons from British Columbia, Alberta, and Saskatchewan utilized publicly available, province-specific databases recording the amount/type of surgeries performed by individual general surgeons [10,11,12]. There is no accepted definition for what designates a general surgeon as being a high-volume breast surgeon. For the purposes of our study, surgeons who had completed ≥5 breast surgeries within a 3-month period were selected to participate in order to focus on clinicians with a regular breast surgical practice. The remaining participants were selected by contacting surgical offices around Canada to obtain contact information for those surgeons regularly performing breast surgeries or because they were known by the authors to have a breast practice. Although this does not admittedly provide a formal representation of each and every general surgeon from all the provinces and territories across the entire country, it will hopefully serve as a snapshot of current practice among a group of general surgeons with a specific interest in breast surgical oncology.

The survey answers were anonymized in order to encourage respondents to give as open and honest a reflection of their practice as possible. The questionnaire was composed of 35 questions and was divided into 6 sections: surgeon/facility background, multidisciplinary rounds, work-up and management, access to plastic surgery, surveillance, and conclusion. The sections ranged from 2–16 questions in length and included a mixture of multiple choice, select all that apply, and short answer questions. The survey was piloted with 11 local surgeons before being sent out to the remaining participants across the country. When the survey closed, the data was collected and analyzed using SurveyMonkey^®^ and Microsoft Excel.

## 3. Results

**Figure 1:**
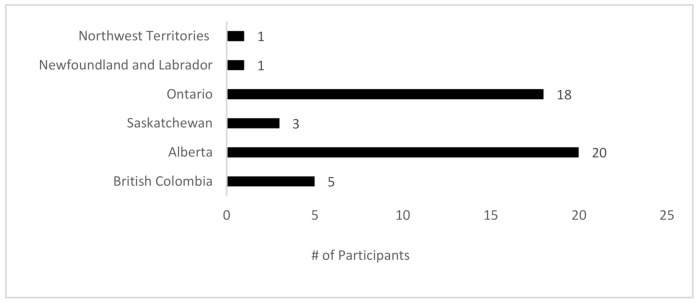
Number and location of those participating in this survey.

A total of 51 (43% response rate) surgeons responded to the survey. Of those, 3 surgeons did not complete the survey in its entirety and their results were subsequently excluded from the analysis. A total of 48 surgeons, therefore, contributed to the findings of this study from select provinces/territories (Figure 1).

### 3.1. Participant Demographics

Of the 48 contributing surgeons, over half (*n* = 28; 58%) classified themselves as general surgeons, with the remainder categorizing themselves as primarily breast surgeons with a component of general surgery emergency call (*n* = 9; 19%), surgical oncologists (*n* = 8; 17%), or pure breast surgeons (*n* = 3; 6%). Participants reported working in independent practice for a median of 15 years (Range 2–40 years), with a mean of 63% (3–100%) of respondents’ annual surgical procedures classified as breast cancer resections.

Over half of survey participants (*n* = 25; 52%) practice in community centers with an academic affiliation, while almost one-third (*n* = 14; 29%) reported practicing in an academic-only setting. The majority of participants indicated they held the title of Assistant Clinical Professor (*n* = 13; 27%), Associate Clinical Professor (*n* = 12; 25%), or Clinical Lecturer (*n* = 13; 27%). The median population of participants’ cities of practice was 800,000 (Range: 10,000–4,000,000). The majority of participants reported completing their medical training within Canada with 7 (16%) respondents indicating that had completed their training abroad.

### 3.2. Indications for Breast Conserving Therapy in DCIS

Questions designed to challenge the usual dogma surrounding indications for mastectomy proved that the vast majority of surgeons polled were keen to pursue breast conservation surgery in clinical scenarios that historically have mandated a mastectomy. 96% (*n* = 46) of respondents would offer BCS for Paget’s disease of the nipple (if radiological workup showed no other abnormalities), all the surgeons polled would offer BCS for multifocal unicentric lesions in a moderate to large sized breast (i.e., two small lesions in the same quadrant), and 96% (*n* = 46) of surgeons would offer BCS for lesions containing a >60 mm area of microcalcification in a large volume breast.

With firmer indications for mastectomy, however, a variation in surgical practice was noted—with 38% (*n* = 18) of respondents preferring to avoid BCS for multicentric lesions and 58% (*n* = 28) preferring to perform a mastectomy for diffuse microcalcifications.

### 3.3. Oncoplastic Breast Surgery

Most participants (*n* = 40; 83%) were able to offer some form of oncoplastic breast surgery (OPBS). Of those who did, 60% (*n* = 24) offered level 2 OPBS. Only 4 (8%) of the 48 surgeons polled, however, are confident in performing the full range of level 2 oncoplastic resections by themselves, with 83% (*n* = 20) performing some or all level 2 OPBS procedures as a combined case with either Plastic Surgery or an oncoplastic-trained colleague.

### 3.4. Sentinel Lymph Node Biopsies

**Figure 2:**
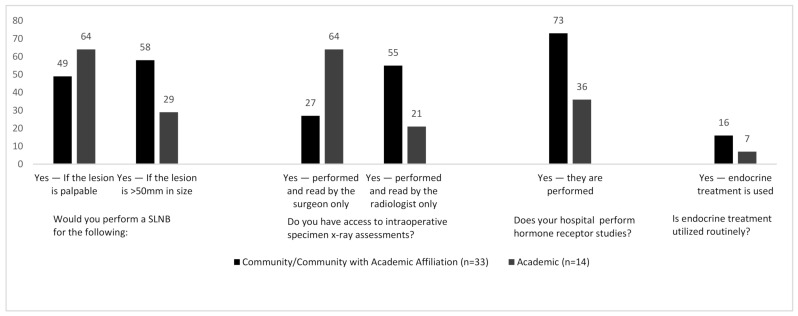
% of participants responding to various questions broken down by site of practice: Community vs. Academic.

The majority of participants in our study adhered to international guidelines with 90% (*n* = 43) of surgeons stating they would perform a Sentinel Lymph Node Biopsy (SLNB) when performing a mastectomy for DCIS. Variation however was seen for other indications: 48% (*n* = 23) of respondents would perform a SLNB for lesions > 5 cm (when comparing surgeons practicing in community and academic centers these figures were 58% and 28% respectively—Figure 2), 52% of surgeons would perform a SLNB for palpable DCIS, and 56% of surgeons would perform SLNB during breast resections which may disrupt breast lymphatics irreversibly—making SLNB almost impossible in the future. As with most survey studies, individual outliers were identified who do not adhere to published evidence: One surgeon stated that they would perform a SLNB during breast conservation surgery for all cases of high-grade DCIS, whilst another suggested that they would perform surgical axillary staging depending on the location of the tumor within the breast. One surgeon performed SLNB routinely during BCS for all DCIS, stating that these lesions are “routinely upgraded to invasive carcinoma”.

### 3.5. Margins

Most participants were able to perform intraoperative X-ray margin assessment (*n* = 40/48). Of those, 18/40 (45%) surgeons read and interpret the intra-operative specimen X-ray themselves, whilst 22/40 (55%) rely on interpretation by the radiologist. This assessment was completed within the radiology department 63% of the time and was completed in the OR for the remainder of cases. There was also a correlation between the size of the town/city and intra-operative specimen X-ray reporting, with surgeons in larger cities being more likely to read their own specimen X-rays. This held true for those practicing in academic centers, where the majority of surgeons performed the assessment themselves as opposed to community settings where the assessment was more likely to be performed by the radiologist (Figure 2).

83% (*n* = 40) of respondents regarded 2 mm as the minimum acceptable margin for DCIS—in keeping with the consensus guidelines for breast conservation therapy. 5 Surgeons (11%), however, suggested that either a 0–1 mm or 1–2 mm margin would be acceptable. 6 respondents alluded to other factors when making a decision on final margin status (such as the size and/or grade of tumor).

### 3.6. Endocrine/Radiation Therapy

Substantial variation was seen with regards to hormone receptor studies in cases of pure DCIS. Nearly two-thirds of total participants (63%) reported that hormone receptor studies are routinely performed for DCIS. Somewhat paradoxically, only 13% of respondents reported that endocrine treatment is routinely used as part of DCIS management. Interestingly, 73% of respondents in community hospitals implied that their pathology departments perform biomarker studies whereas only 36% of those from academic centers perform biomarker analysis (Figure 2). One respondent indicated that biomarkers are performed on all DCIS lesions measuring >2 cm. Another respondent alluded to the current global COVID-19 coronavirus pandemic and suggested that all biopsies containing pure DCIS are undergoing biomarker studies, so as to commence endocrine therapy, due to the current potential limitations being placed on operating rooms and hospital resources.

When counseling on the need for adjuvant whole breast radiotherapy in patients with DCIS, two-thirds (*n* = 32; 67%) of participants indicated that they take absolutely no role in the decision-making process for adjuvant whole breast radiation. The size of the tumor, margin width, grade of tumor, and age of the patient were all factors that were taken into account when recommending radiotherapy by around 35–40% of respondents.

### 3.7. Surgical Volume

**Figure 3:**
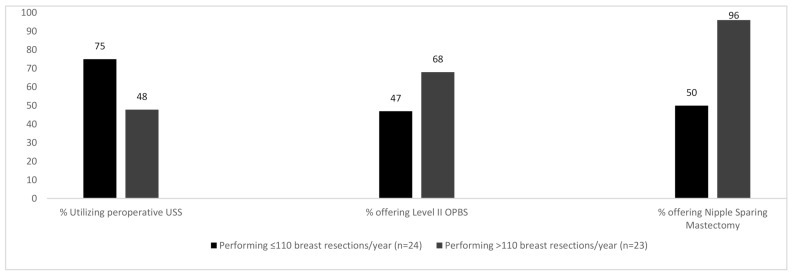
% of participants responding to various questions broken down by volume of annual breast resections.

The number of breast procedures performed annually by participants heavily influenced the outcomes of the questions posed. A median of 110 breast resections was performed by all participants. Surgeons performing >110 breast resections/year were more likely to offer BCS for multicentric disease compared to their counterparts who treated fewer patients and a larger percentage of these high-volume breast surgeons were also able to offer Level II OPBS (68% vs. 47%) and nipple sparing mastectomy (96% vs. 50%)—(Figure 3).

75% of surgeons performing a lower volume of breast cases per year reported using breast ultrasound assessment preoperatively (which is not supported by practice guidelines). Conversely, higher volume breast surgeons report using pre-operative US only 48% of the time. ‘Low-volume’ breast surgeons were more likely to have their intraoperative specimen X-rays performed and read by a radiologist whereas ‘high volume’ breast surgeons were more likely to perform and read intra-operative X-rays themselves.

### 3.8. Follow-Up and Surveillance

All surgeons who responded to this survey agreed that their patients should undergo mammographic screening, at least in the short term. The majority (*n* = 34; 71%) of respondents confirmed a protocol of annual mammographic screening for life for patients who have completed treatment for DCIS. Consensus was lacking in the length of this radiological follow-up with 17% of surgeons performing mammographic screening annually for 5 years and 4% for 10 years following treatment. Furthermore, agreement was lacking in the length of surgical follow-up with almost half (48%) of responding surgeons choosing not to follow-up their patients after treatment for DCIS beyond 1 month. 29% of surgeons see patients for up to 1 year and 15% follow-up for up to 5 years.

## 4. Discussion

This study—which represents a limited snapshot of surgical practice across Canada—demonstrates substantial variation in the surgical management of DCIS, with marked differences in the routine imaging workup, intra-operative decision-making, and follow-up care for patients with DCIS.

Variation in the management of DCIS has been demonstrated previously at both national and international levels including various ongoing studies regarding the optimal management of this disease [7,8,13,14]. A study looking at variation in practice in the UK, for example, demonstrated national differences in the rates of, and indications for, mastectomy, axillary management, and follow-up procedures for DCIS patients [15,16,17]. These international uncertainties regarding the optimal management of DCIS offer a possible explanation for the lack of consistency regarding its management across Canada. An additional explanation for part of this variation relates to the presence of plastic surgeons in rural locations or the distance to tertiary/quaternary centers where complex breast reconstructive procedures can be performed. Combined, these limitations and uncertainties indicate the need to create Canadian-specific guidelines or, at the least, encourage increased adherence to NCCN guidelines.

Our survey confirmed unanimity regarding the use of breast conserving surgery for patients with multifocal unicentric lesions, but there was substantial variation for firmer indications for a mastectomy, such as multicentric lesions and diffuse microcalcifications on imaging. There was also marked variation regarding access to nipple sparing mastectomy and immediate breast reconstruction.

This variation in breast sub-specialization and individual surgeon volumes leads to an interesting debate with regards to the future of breast surgery. It could be argued that offering Oncoplastic Breast Surgery and Nipple-Sparing Mastectomy (NSM) is a surrogate marker for contemporary practice and a sub-specialist skillset. Interestingly, surgeons offering NSM (with access to immediate breast reconstruction) were more likely to attend weekly multidisciplinary breast tumor-board meetings and offer their patients a surveillance-only strategy for low-risk DCIS in keeping with the currently ongoing COMET, LORIS, and LORD prospective trials. Moreover—although it wasn’t specifically investigated in this study—surgeons with a highly sub-specialized high-volume breast surgical practice are more likely to attend annual international breast surgery conferences and educational meetings, and therefore be more up-to-date with contemporary data, current trends, and ongoing trials within breast oncology. This finding adds weight to published data showing a trend towards better surgical and oncological outcomes in higher volume hospitals, including a higher rate of breast conservation in these higher volume centers [18,19].

This study also shows some variation in the use of surgical axillary staging when treating DCIS, with approximately half of the surgeons polled offering SLNB to patients with biopsy-proven DCIS when the lesion is palpable or if the size exceeded 50 mm. Axillary staging for DCIS remains controversial. National and international guidelines advise against axillary staging during breast conservation surgery [20]. Despite this, anecdotal evidence suggests that a disproportionate number of surgeons continue to perform sentinel lymph node biopsy for in-situ disease. One respondent to our survey justified axillary SLNB suggesting that all DCIS harbors a risk of upgrade to invasive disease—a statement with no basis within the published surgical literature [21]. The NCCN guidelines support SLNB for DCIS only in the setting of a mastectomy, or during resection of an anatomic location that may compromise the performance of a future sentinel node procedure [20]. Formal guidelines from the American Society of Breast Surgery on the role of SLNB during oncoplastic breast surgery for DCIS is awaited, but an analysis by Pyfer et al. analyzing the American College of Surgeons’ National Surgical Quality Improvement Program (ACS NSQIP) data showed a non-adherence to national guidelines in 20–30% of cases [22].

Of more interest are the marked differences in practice observed regarding the minimum acceptable margin width for pure DCIS, despite international consensus guidelines recommending negative margins of at least 2 mm. The seminal meta-analysis by Houssami et al. suggested that a 2 mm margin resulted in a significant decrease in the likelihood of local recurrence (by up to 50%) when compared to patients with positive margins (defined as “tumor on ink”) [20,21]. The majority of participants polled within our study adhered to these guidelines but more than 1 in 10 surgeons reported routinely accepting margins < 2 mm—further justifying the need for the development of national specific guidance on margin assessment.

Hormone receptor studies are yet another source of geographical variation—not only within Canada but globally [16,23]. The American Society of Clinical Oncology and College of American Pathologists (ASCO/CAP) guidelines suggest that “*Testing of DCIS for ER is recommended to determine potential benefit of endocrine therapies to reduce risk of future breast cancer, while testing DCIS for PgR is considered optional*” [24]. Whilst European guidelines do not recommend adjuvant endocrine treatment for in-situ disease when managed with BCS and radiotherapy, citing no survival benefit, the US-based NCCN guidelines, support the routine use of adjuvant anti-estrogens for ER-positive DCIS—even when managed with BCS and radiation treatment. 63% of surgeons polled in this survey indicate that ER-receptor studies are routinely performed on their DCIS specimens [25]. Interestingly, however, only 12% of respondents implied that their multidisciplinary team employs adjuvant endocrine treatment in their practice. Aside from the obvious financial impact of performing this unnecessary testing, the result of which is not routinely acted upon, this serves as further corroboration of the need for standardization of practice across Canada so as to offer patients the same investigations and treatment no matter where they live [20]. Additionally, a recent study conducted in British Columbia found that the utilization of endocrine treatment for patients with DCIS varied according to the treatment center (8–13%) as well as the prescribing oncologist (0–40%) [14]. At the very least, in the era of ‘personalized medicine’, we propose the need for a more targeted approach to biomarker testing in order to optimize patient care at an individual level [7]. In real world practice there is an interplay between three specialties (general surgery, medical oncology, and radiation oncology) when it comes to the prescribing of endocrine treatment. In the U.K. for example, both general surgeons and oncologists routinely prescribe anti-estrogens, whereas in Canada it is more commonplace for oncologists to do so.

Two thirds of respondents admitted taking absolutely no part in the decision-making process around the need for adjuvant radiation. As we move further towards a model of breast sub-specialization—and as the idea of ‘shared decision-making’ gains speed—one has to question whether the onus lies with the surgeon to take patients through a full and detailed discussion of the factors contributing to the need for whole breast radiation. Without such a conversation, one must question whether patients with little to no medical knowledge can truly make an informed decision regarding their surgical option of choice. Furthermore, patients with low-risk DCIS (e.g., small, low grade lesions) may be able to avoid radiation after breast conservation surgery altogether due to their inherent low risk of local recurrence [26,27].

Oncoplastic breast conservation surgery has long been standard of care in Europe as well as other parts of the world. We have previously published on the relative lack of Oncoplastic training opportunities in North America resulting in lower rates of OPBS [28]. A recent Canadian study from Ontario advocates for greater access to OPBS fellowships for Canadian surgeons to provide a wider experience/practice and in turn allow them to offer Level II OPBS to a larger percentage of patients with breast cancer [17]. Despite this, only 8% of surgeons polled in our study are able to offer their patients the full range of oncoplastic options without the need for a combined approach with plastic surgery. This further justifies the need for a shift towards a more breast sub-specialist training model as well as increasing the number of—and access to—oncoplastic teaching opportunities within Canada.

Access to a breast multidisciplinary meeting seems consistently high with all the Canadian surgeons polled in this study. When in place, Multi-Disciplinary Teams (MDTs) provide an opportunity for surgeons, oncologists, pathologists, and radiologists to formulate patient- and tumor-specific management plans, communicate with patients and other members of the healthcare team, and make decisions regarding further investigations [29]. Where implemented, MDTs have resulted in increased adherence to standardized guidelines [30]. Furthermore, this adherence to guidelines has been shown to optimize patient outcomes, reduce the number of perioperative complications, and decrease the patient’s length-of-stay in hospital [31,32,33]. Additionally, increased adherence to guidelines has also been shown to increase job satisfaction and increase perceived levels of physician autonomy [34].

There is little doubt that some patients experience unnecessary morbidity from the aggressive overtreatment of this disease when compared to the relatively indolent nature of the pathology and could benefit from less aggressive treatment methods [35]. Learning lessons from the urological community after seeing a profound shift in the management of certain low Gleason-score prostate cancer to an “observation-alone” strategy, the LORD, LORIS, COMET, and LORETTA trials are currently underway and will strive to provide an evidence-base, supporting a surveillance-alone strategy in select cases of non-high-risk DCIS [8,13]. Our results show substantial support amongst Canadian breast surgeons for the adoption of surveillance-alone for certain types of DCIS both now and once this evidence is published. Future guidelines should be sympathetic to Canadian surgeons so that they may adapt their current practice in order to better balance the risk/benefit ratio of surgical (and adjuvant) treatments. The end result of these surveillance trials may well see a future major reduction in the rate of surgical intervention in certain subsets of low-risk DCIS.

One of the drawbacks of this type of survey study is the inherent selection bias that can occur. It can be argued that surgeons with a specific interest in breast surgical oncology, who therefore stay up-to-date with current trends and guidelines in the management of DCIS, have a vested interest and are more likely to respond to such a study compared to their counterparts who view breast surgery as only a small part of their surgical practice. Therefore, the answers, results, and conclusions drawn from survey-type studies may be a misrepresentation of national surgical practice as a whole. As we move further towards breast subspecialization, however, it could be counter-argued that high-volume breast surgeons are exactly the group of clinicians that should be scrutinized for this type of study, as they benefit from a large throughput of clinical volume and will be instrumental in defining future practice with national educational meetings and guideline formulation.

Although our study indicates substantial variation in the various aspects of the management of DCIS, our sample size is moderate and does not include surgeons from all provinces and territories. This needs to be taken into consideration when drawing far-reaching, practice-changing conclusions. Additionally, while the survey was sent out to surgeons in nearly every province, the majority of the responses were from Alberta and Ontario and might therefore reflect a biased sample. Furthermore, there may be differences in clinical practice vs. the individual responses. Regardless, the trends and responses observed in this study provide a valuable snapshot into the current surgical practice surrounding DCIS across Canada, suggesting the need for the development of evidence-based pan-Canadian guidelines.

## 5. Conclusions

This study, despite a limited sample size, suggests a marked variation in the surgical management of DCIS within Canada. Variation was most apparent in access to more complex oncoplastic procedures, the indications for performing SLNB, and in biomarkers studies/hormone therapy. The development of national guidelines should aim to minimize variations in practice, whilst identifying areas for future research. Furthermore, the utilization of MDTs will be crucial to decide the best surgical (or non-surgical) strategy to treat this disease.

## Data Availability

The data presented in this study are available upon request of the corresponding author.
